# *In vitro* evaluation of crosslinked bovine pericardium as potential scaffold for the oral cavity

**DOI:** 10.3233/BME-230027

**Published:** 2023-11-10

**Authors:** Guadalupe del Carmén Ordóñez-Chávez, Nayeli Rodríguez-Fuentes, Ricardo Peñaloza-Cuevas, José Manuel Cervantes-Uc, Luz Eugenia Alcántara-Quintana, Ixchel Araceli Maya-García, Virginia Aurora Herrera-Valencia, Celia Elena Mendiburu-Zavala

**Affiliations:** a Universidad Autónoma de Campeche, Campeche, Mexico; b Universidad Autónoma de Yucatán, Yucatan, Mexico; c CONACYT-Centro de Investigación Científica de Yucatán, Yucatan, Mexico; d Centro de Investigación Científica de Yucatán, Yucatan, Mexico; e CONACYT-Universidad Autónoma de San Luis Potosí, San Luis Potosi, Mexico

**Keywords:** Bovine pericardium, dental pulp stem cells, guided bone regeneration, oral cavity

## Abstract

**BACKGROUND:**

Bovine pericardium (BP) is a scaffold widely used in soft tissues regeneration; however, its calcification in contact with glutaraldehyde, represent an opportunity for its application in hard tissues, such as bone in the oral cavity.

**OBJECTIVE:**

To develop and to characterize decellularized and glutaraldehyde-crosslinked bovine pericardium (GC-BP) as a potential scaffold for guided bone regeneration GBR.

**METHODS:**

BP samples from healthy animals of the bovine zebu breed were decellularized and crosslinked by digestion with detergents and glutaraldehyde respectively. The resulting cell-free scaffold was physical, chemical, mechanical, and biologically characterized thought hematoxylin and eosin staining, DNA quantification, scanning electron microscopy (SEM), Fourier transform infrared spectroscopy (FTIR), thermogravimetric analysis (TGA), differential scanning calorimetry (DSC), uniaxial tensile test, cell viability and live and dead assay in cultures of dental pulp stem cells (DPSCs).

**RESULTS:**

The decellularization and crosslinking of BP appeared to induce conformational changes of the CLG molecules, which led to lower mechanical properties at the GC-BP scaffold, at the same time that promoted cell adhesion and viability of DPSCs.

**CONCLUSION:**

This study suggests that the decellularized and GC-BP is a scaffold with the potential to be used promoting DPSCs recruitment, which has a great impact on the dental area.

## 1. Introduction

According to the World Health Organization, dental cavities and periodontal disease are the most prevalent oral problems that affect more than 90% of the population [1]. The progression of these diseases causes dental loss that leads to bone resorption. For this reason multiple scaffolds have been designed for bone regeneration in oral cavity [2–5]. To do this, several scaffolds manufactured using natural or synthetic materials have been used to promote the migration of pluripotential and osteogenic cells to the bone defect site [2,3,6–15].

In this context, the bovine pericardium (BP) which has been used as natural scaffold to repair cardiac defects [16,17], has been reported to be susceptible of calcification in contact with crosslinking reagents like glutaraldehyde (GA) [18,19], reason by which, BP has been analyzed in various bone regeneration studies both *in vitro* and *in vivo*, native, crosslinked or decellularized [14,20–22].

The participation of BP in the process of repair or bone regeneration has been accompanied by the development of various methods to preserve its ECM and decrease or elimination of cellular and genetic material [23–26]. For this purpose, often is used the decellularization process because is an effective way to remove the cellular material, preserve the ultrastructure, and maintain the mechanical anisotropy and tensile properties of BP [23,27–29]. In light of the findings suggesting that the decellularization process decreases calcification, while cross-linking with GA promotes it, this work focused in obtaining the BP scaffold through the decellularization process and further crosslinking with GA for its potential application in bone regeneration of oral cavity. The scaffold obtained was characterized at the physicochemical, mechanical and biological level. The cytotoxicity was evaluated using *in vitro* assays with dental pulp stem cells (DPSCs), since it has been demonstrated that these cells participate in tissue regeneration in bone of oral cavity [30,31]. The development of a scaffold from a bovine model will offer an opportunity in tissue engineering to be used in bone regeneration in the oral cavity or as a scaffold to promote healing after tooth extraction, due the decellularization and glutaraldehyde-crosslinking of BP, improved the physicochemical and mechanical properties of BP, preserved the ECM and promoted the adhesion and cell viability of DPSCs.

## 2. Materials and methods

### 2.1. Materials

Reagents used in cell culture were obtained from Caisson Laboratories (USA). Cell Titer-Blue Viability Reagent was purchased from Promega Corporation (USA). Reagents for molecular biology, paraformaldehyde (PFA) and glutaraldehyde (GA) were delivered by Sigma-Aldrich (USA). Crystal violet was acquired from Hycel (Mexico). The CD90-FITC, CD105-PE-Cy5, CD73-APC, CD34-PE and CD45-FITC markers, and Trucon tubes were purchased from BD Bioscience (USA).

### 2.2. Decellularization and crosslinking of bovine pericardium (BP)

BP samples were collected from healthy animals of the bovine zebu breed (male, ranging in weight of 1700 lb to 2200 lb) from a local slaughterhouse with register Federal Inspection Type at Mérida, Yucatán, México. Samples of BP with an area of 20 cm^2^ were cut with a scalpel and washed three times with phosphate-buffered saline (PBS) solution containing 1% of antibiotic-antimycotic. Then, the samples were decellularized according to Gardín [21]. The crosslinking of the decellularized BP (Dcell-BP) was performed thought the incubation of samples in a 0.5% (v/v) GA solution at 4 °C, pH 7 for 15 days. After that, scaffolds were washed twice with ethanol solution 10% (w/v) for 1 h at 25 °C to obtaining decellularized and GA-crosslinked BP (GC-BP). The effectiveness of the decellularization protocol was determined qualitatively by histology and eosin-hematoxylin staining (H&E) as previously reported [32]. Additionally, the residual deoxyribonucleic acid (DNA) measurement was performed on n-BP, Dcell-BP and GC-BP (200 mg) as reported previously [5,33], using a spectrophotometer (Nanodrop 2000, Thermo Fisher Scientific Inc., MA, USA). To determine DNA fragment size, 10 μg of DNA of each sample were resolved by electrophoresis in 3% (w/v) agarose gel, with Ethidium Bromide at 60 V for 1 h; and revealed with ultraviolet transillumination in a Gel Doc Ez Imager (Bio-Rad, CA, USA).

### 2.3. Physicochemical characterization

The microstructure was analyzed with a scanning electron microscope JEOL 6360 LV with a voltage of 20 kv. For this, samples (disks of 5 mm diameter) of n-BP and GC-BP were fixed in GA solution (0.5%) for 30 min, at 4 °C and gradually dehydrated in ethanol solutions (10% to 100% v/v). Subsequently, the samples were gold coated with a Denton Vacuum, model Desk II equipment (NJ, USA) and placed directly on the sample holder.

Fourier transform infrared (FTIR) analysis was realized in Thermo Scientific Nicolet TM 8700 FT-IR spectrometer (MA, USA), with a wave number range of 4000–650 cm^−1^. The samples of 10 mg were placed directly in the spectrometer and analyzed with ATR/ZnSe crystal, using 100 scans and a resolution of 4 cm^−1^ [34,35].

The thermal behavior was studied by thermogravimetric analysis (TGA) and by differential scanning calorimetry (DSC). TGA curves were obtained with a TGA 8000 from Perking Elmer Company (MA, USA) by heating 10 mg of the freeze-dried (Labconco FreeZone 1, MO, USA) samples from 50 °C to 700 °C, at a heating rate of 10 °C/min under a nitrogen atmosphere as described by previous studies [36,37]. DSC thermograms were obtained with DSC-7 Instrument (Perkin Elmer Company, MA, USA). For this, 10 mg of the scaffold were place in aluminum pans and heated from 0 °C to 300 °C at a heating rate of 10 °C/min under inert atmosphere.

The mechanical properties of samples were determined by uniaxial tensile test; for this, six rectangular tissue specimens were used. Specimens with dimensions of 60 × 10 × 10 mm from n-BP and GC-BP tissues were obtained with a scalpel. The assays were performed using a Mini Shimadzu universal testing machine, model AGSX (BioSpec, Honk Kong, China) with a 10.0 N load cell (type S). The programmed displacement rate was 5.0 mm/min and 0.01 N was applied before the test [38,39].

### 2.4. Biological characterization

DPSCs were obtained from third molars of male patients who attended at the Oral Surgery Clinic of the Autonomous University of Yucatan, Mexico, and signed the consent. The protocol was approved by the ethics committee from Dr. Hideyo Noguchi Regional research Center (CIE-06-2017) and was carried out in accordance with the Declaration of Helsinki. DPSCs were isolated by explant technique as previously reported [40,41]. For this, each tooth was fractured at the amelocementary limit with a micromotor Ti-Max X205L and a diamond disc in order to expose the pulp, the this tissue was fragmented into explants with a scalpel. The pulps fragments were incubated at 37 °C under a humidified atmosphere containing 5% CO_2_ in DMEM supplemented with penicillin (100 mg/mL) streptomycin (100 mg/mL) and 10% of FBS until reaching 80% confluence.

Cell characterization was performed with cells from 4th passage through multipotency (osteogenic and adipogenic differentiation), clonogenicity (units forming colonies assay) and immunophenotypic profile (positivity towards surface markers such as CD90-FITC, CD105-PE-Cy5, CD73-APC, CD34-PE and CD45-FITC) as previously reported [42]. Additionally, the dental pulp structure was observed by histological analysis using toluidine blue staining.

To evaluate the ability of GC-BP scaffolds to promote the cell viability, DPSCs were cultured on n-BP and GC-BP scaffolds (5 mm of diameter) at a cell density of 1.5 × 10^3^ in a 96-well plate for 24 h; after this time, 20% of Cell Titer-Blue reagent was added and incubated for 4 h in cell culture standard conditions according to the manufacturer’s instructions. Additionally, 5 × 10^3^ cells were cultured on either n-BP or GC-BP disks (5 mm of diameter) at 37 °C, under cell culture standard conditions, for 24, 48 and 72 h. Then, cell cultures were stained with the Live/Dead assay reagents according to the manufacturer’s protocol and analyzed with Olympus Fv1000 confocal microscope (Olympus America de Mexico, Mexico), using a high-voltage laser at 488 nm and 615 nm excitation for EthD and CAL, respectively.

### 2.5. Statistical analysis

Statistical analysis was performed using Origin v.10 (Northampton, MA, USA.). All results are expressed as mean ± standard deviation of 5 repetitions. Parameters were compared using ANOVA with subgroup (BC, GC-BP and control groups) analyses using Bonferroni correction and Tukey test for multiple testing; a *p*-value less than 0.05 was considered statistically significant.

## 3. Results

### 3.1. Decellularization efficiency of bovine pericardium

**Fig. 1.**
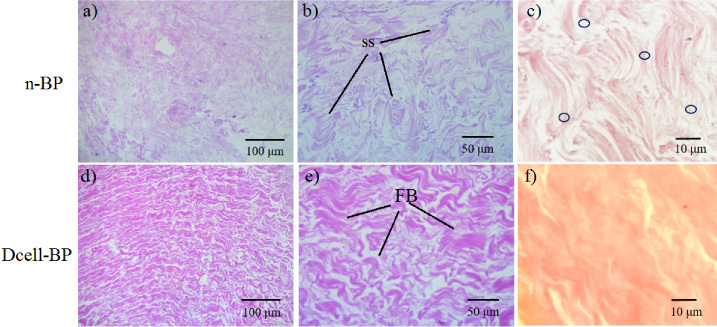
Histological analysis of the decellularization process. Samples were processed with hematoxylin and eosin staining. Native bovine pericardium, n-BP (a–c) and the bovine pericardium decellularized, Dcell-BP (d–f). SS indicates CLG arranged in a swirling structure; the circles show cell nucleus. FB indicates CLG in bundles (eosinophil material arranged in parallel bands with each other).

**Fig. 2.**
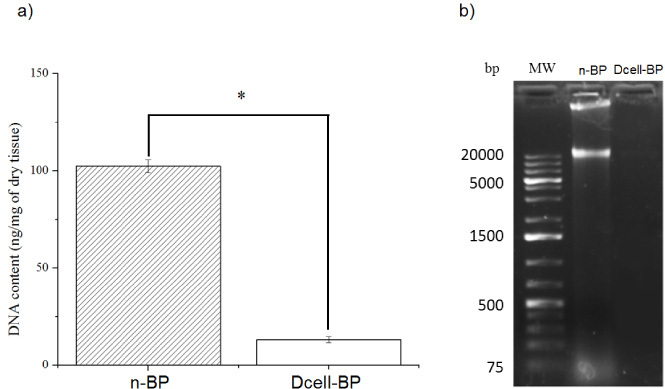
DNA remaining determination. (a) Quantification of DNA by absorbance, (b) Gel electrophoresis of DNA isolated from native bovine pericardium (n-BP) and decellularized bovine pericardium (Dcell-BP). MW, molecular weight in base pairs (bp). *n* = 5, *p < 0.05.

We obtained decellularized BP (Dcell-BP), which was crosslinked with glutaraldehyde (GC-BP). The effectivity of the decellularization process on the BP structure and cell residual content was evaluated by eosin-hematoxylin staining (Fig. 1). Preservation of ECM structure and a decrease in cell content for Dcell-BP in comparison with n-BP were observed after the decellularization process. A typical mesenchymal tissue was observed in n-BP, which also shown CLG fibers with a swirling structure (Fig. 1a, b) likewise, there are elongated nuclei compatible with fibroblasts (Fig. 1c). In Dcell-BP, well-organized CLG fiber bundles were observed (Fig. 1d, e), showing a dense connective tissue and no cell debris (Fig. 1f). Also, significant differences were observed in the genetic material content for n-BP and Dcell-BP samples (Fig. 2). n-BP showed a DNA content of 102.4 ± 3.3 ng/mg dry weight, while the Dcell-BP had a 13.1 ± 1.6 ng/mg dry weight, which represented a decreased of 87.2% of DNA content (Fig. 2a) so it can be considered as decellularized scaffold in according to previous reports [43]. The DNA fragments in n-BP were identified by gel electrophoresis; these showed a high molecular weight of approximately 20,000 base pairs, whereas DNA fragments were non-detectable in Dcell-BP scaffold (Fig. 2b).

### 3.2. Crosslinking and physicochemical properties of GC-BP

**Fig. 3.**
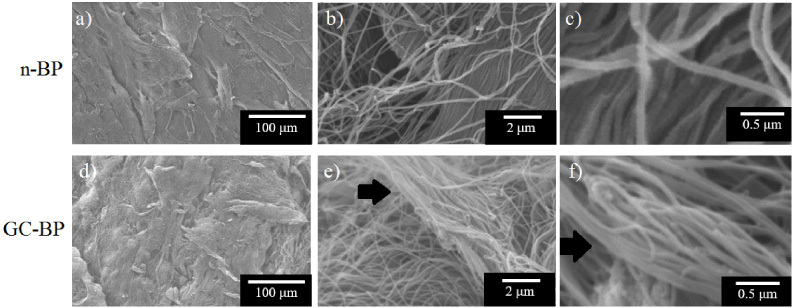
SEM analysis. Micrographs of native pericardium (n-BP) in (a–c). Decellularized and crosslinked glutaraldehyde bovine pericardium (GC-BP), is shown in (d–f). The black arrows show the conglomerates of collagen fibers.

Due to the fact that our objective was to obtain a decellularized and cross-linked scaffold, and to evaluate its physicochemical, mechanical and biological characteristics, the subsequent analyzes were carried out comparing the n-BP with the cross-linked and decellularized one, GC-BP scaffold, so the effect of only decellularization (Dcell-BP) or only crosslinking were not considered. In this context, the SEM analysis was made to observe the microstructure of GC-BP vs n-BP, this analysis showed slight alterations in the CLG fibrillary arrangement of the native sample after decellularization and crosslinking processes (Fig. 3). In the panoramic vision at 100 μm (Fig. 3a, d) it was possible to observe a laminated morphology in both samples (n-BP and GC-BP); however, in a closer inspection, an arrangement of isolated CLG fibers was observed in the n-BP (Fig. 3b, c) while for GC-BP some fibers conglomerates were observed (Fig. 3e, f).

**Fig. 4.**
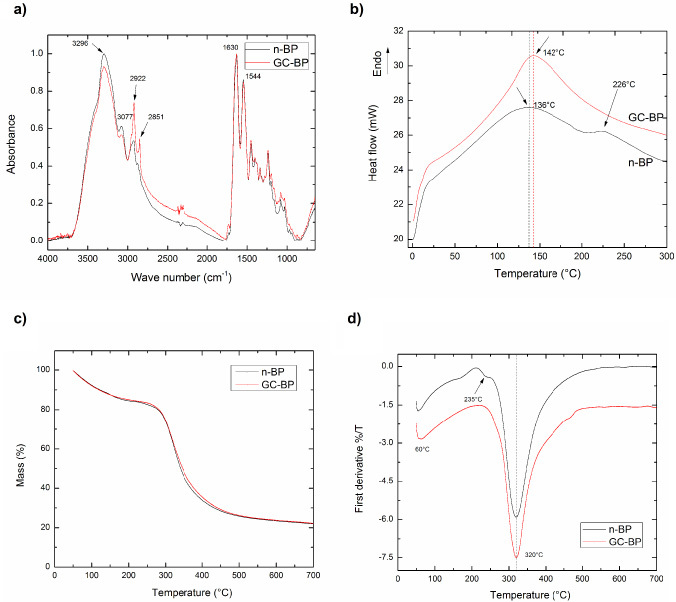
Physicochemical characterization of scaffold. (a) FTIR Spectra, the spectra was normalized with respect to the band of a maximal intensity in amide I region (1630 cm^−1^). (b) DSC thermograms, ENDO shows the endothermic orientation. (c) TGA, (d) DTGA. Black line and red line represent the n-BP and GC-BP, respectively. The arrows indicate the maxim point in the spectra.

Figure 4a shows the FTIR spectra of both n-BP and that obtained after the decellularized and crosslinked processes, GC-BP. It can be seen that spectra show bands at 3296 cm^−1^, due to the N-H stretching vibration for the amide A, and at 3077 cm^−1^, associated to C-H stretching for amide B [34,44]. Doyle pointed out that these bands are related to N-H stretching vibration and amide II overtone. The bands at 2922 cm^−1^ and 2851 cm^−1^ correspond to the asymmetric and symmetric C-H stretching vibrations of methylene groups. Spectra also display a band at 1633 cm^−1^ which has been related to overlapping of C=O stretching, C-N stretching and N-H bending vibrations, whereas the peak at 1544 cm^−1^ was attributed to N-H bending and C-N stretching vibration [44,45].

DSC thermograms for n-BP and GC-BP scaffolds are shown in Fig. 4b. An endothermic transition was detected at 136 °C and 142 °C for n-BP and CG-BP respectively, which was attributed to release of water from samples. An additional endothermic peak at 226 °C was detected in n-BP but not in CG-BP.

Thermal decomposition behavior of n-BP and GC-BP is shown in Fig. 4c–d. As observed, the thermal decomposition behavior of the n-BP was very similar to that displayed by BP-GC as both samples showed two degradation stages; the first one was located at 60 °C whereas the second one was observed at 320 °C. A small signal at 235 °C was detected in n-BP but not in GC-BP sample; residual masses for both samples were around 20 wt%.

### 3.3. Mechanical properties of GC-BP

The ultimate tensile strength, Young’s modulus and elongation at break were calculated from the experimental stress–strain curves and the obtained results showed for GC-BP a lower Ultimate Tensile Strength (UTS) values (0.54 ± 0.10 MPa) in comparison to n-BP (0.82 ± 0.24 MPa). A similar behaviour was displayed by Young’s Modulus, which decreased after the treatment from 9.11 ± 3.58 for n-BP to 4.44 + 1.07 for GC-BP. In contrast, GC-BP showed higher deformation than that exhibited by native tissue; exhibiting values of 35.57 ± 8.77 and 18.86 ± 4.28 of Elongation at break, respectively (Table 1).

**Table 1:** Tensile mechanical properties of n-BP and GC-BP

Scaffold	Ultimate tensile strength (MPa)	Young’s modulus (MPa)	Elongation at break (%)
n-BP	0.82 ± 0.24	9.11 ± 3.58	18.86 ± 4.28
GC-BP	0.54 ± 0.10	4.44 ± 1.07	35.57 ± 8.77

*n* = 6.

**Fig. 5.**
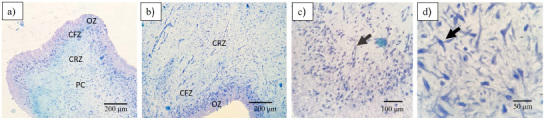
Histological examination of dental pulp. (a) It can be distinguished 4 zones of dental pulp: OZ = Odontoblastic Zone, CFZ = Cell Free Zone, CRZ = Cell Rich Zone and PC = Pulp core. (b) Can observed odontoblasts (black arrow). (c) In cell zone can observe endothelial cells. (d) Fusiform aspect of dental pulp cells.

**Fig. 6.**
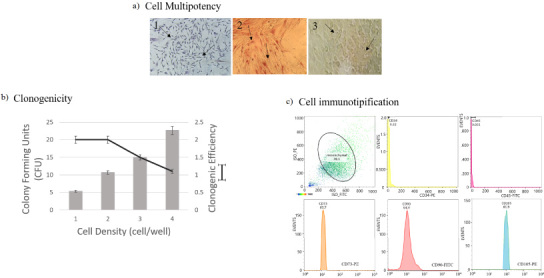
Dental pulp stem cells characterization. (a) Multipotency assay, 1 = basal culture medium, 2 = osteogenic culture medium, 3 = adipogenic culture medium; (b) Clonogenic and clonogenicity efficiency, (c) Immunotipification against CD90-FITC, CD105-Percp-Cy, CD73-APC and CD34-PE/CD45-FITC.

### 3.4. In vitro biocompatibility of GC-BP

**Fig. 7.**
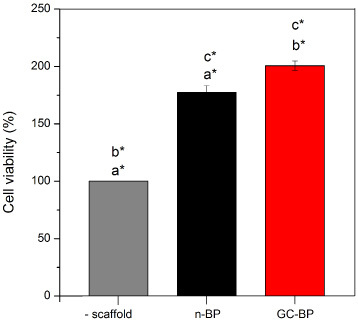
Cell viability on n-BP and GC-BP. Cell viability was measured through absorbance detection at 570/600 nm and reported as percentage respect to control condition consisted of cells cultured on plate dish without scaffold (-scaffold). The same letters indicate statistical comparison between experimental conditions. *p < 0.05, *n* = 3.

The health and integrity of the pulp tissue were histologically analyzed. In Fig. 5 four main layers of dental pulp can be observed: the odontoblastic zone (OZ), formed by secretory odontoblasts; the cell free zone (CFZ), identified as an area without cells, also known as oligocellular zone and, the cell rich zone (CRZ) constituted by defensive cells (macrophages, lymphocytes or plasma cells), blood capillaries, nerves; and pulp core (PC), characterized by its high cell density, where mesenchymal cells and fibroblasts stand out, surrounded by abundant ECM. DPSCs obtained for this tissue, were adhered to cell culture plate, showed fibroblast-like morphology and were positives for mesenchymal parameters (Fig. 6). The cells exhibited both adipogenic (positive staining to oil red) and osteogenic differentiation capacity (positive staining to alizarin red) (Fig. 6a). In addition, cells exhibited the ability to generate colony forming units (CFU) at various cell concentrations, which indicated its clonogenic capacity. At low concentrations of cells, it was possible to observe the CFUs. Meanwhile at higher cell concentrations the cells reaching confluence caused lower clonogenic efficiency, which is a typical behaviour of mesenchymal stem cells (Fig. 6b). The cell immunotyping by flow cytometry demonstrated that cells obtained from dental pulp were positive for mesenchymal antigens with total expression of 78%. Figure 6c, shows that cells were 83.7% positive for CD73, 64.4% for CD90, and 81.9% for CD104, meanwhile, the positivity for hematopoietic markers was very slow (0.32% for CD34 and 0.03% for CD45), indicating that it is a cell culture of mesenchymal cells and not hematopoietic cells. Both scaffolds n-BP and GC-BP promoted an increase in cell viability at 24 h in comparison with the cultures without scaffold (100%) as shown in Fig. 7. In addition, the GC-BP induced an increase in cell viability (200.6 ± 4.2%) vs cells cultured on n-BP scaffold (177 ± 5.8%).

**Fig. 8.**
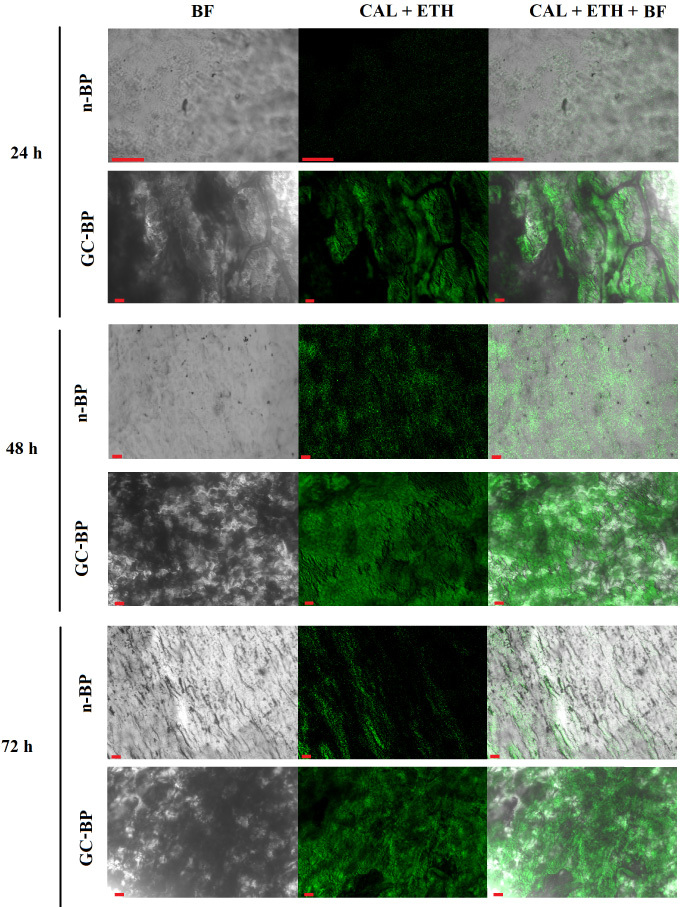
Live/Dead assay of DPSCs by fluorescence. Confocal micrographs of DPSCs exposed to native (n-BP) and decellularized and crosslinked BP (GC-BP). CAL, calcein, EthD, ethidium, BF, bright field. Red scale bar 50 μm.

The Live/Dead assays showed cells emitting an intense green fluorescence confirming the cell viability in both scaffolds (n-BP and GC-BP) as it can be seen in Fig. 8. Interestingly, the images of cells exposed to GC-BP showed a higher amount of adhered cells compared to the n-BP. In the same way, the cell population increased as the exposure time increased in both scaffolds, showing a higher cell population at 72 h of incubation, which suggested that the scaffolds are suitable for cell adhesion and proliferation of DPSCs.

## 4. Discussion

The development of scaffolds is important due to their ability to promote and contribute to the process of tissue regeneration [2,3], in this regard, various materials have been used to make tissue regeneration a reality.

In the field of dental tissues regeneration, the efforts are mainly focused on bone tissue as it provides support and coordinates the spatial distribution of other oral tissues, and even actively participates in dental eruption and in processes such as chewing. In order to carry out the regeneration of the bone of the oral cavity, materials that promote GBR are required, which must allow the growth of the bone while serving as physical barriers that prevent the growth of connective tissue and epithelial tissue within the bone defects [4,5].

Regarding materials with potential application in GBR, the BP has shown to be a viable option due its ability to calcify when is crosslinked with GA [18,46] and because its physicochemical properties improved by GA crosslinking. However, there is evidence that decellularization decreases calcification of BP bioprosthetic valves as previously reported by Collatusso et al. [28]. In this work, we obtained a decellularized and GA crosslinked scaffold from BP (GC-BP). The decellularization process not only showed a significant decrease in the original cell population but also preserved the ECM structure as shown by H&E staining. The matrix of Dcell-BP showed a wavy pattern with multiple layers of the CLG fiber bundles and absence of cell nuclei in concordance with previous studies [39]. The wavy pattern of GC-BP has been related to the promotion of cell proliferation and viability [22–25].

Also, the reduction of DNA content in the Dcell-BP scaffold was higher than that reported by Heuschkel et al. [25], who reached a 77% of DNA reduction with a decellularization process that did not use protease inhibitors. Nevertheless, the percentage of decellularization obtained in the present study was lower than those reported by Mendoza et al. [29] and Zouhair et al. [23], who reported 97% (remnant DNA 75 ng/mg) and 97.5% (remnant DNA 37.8 ng/mg), respectively, although the remaining DNA obtained in this work was lower (remaining DNA 13.1 ng/mg) than the obtained by those authors. In this regard, Zouhair et al. used sodium dodecyl sulphate and alkaline surfactants whereas in the present work, we used Triton X100, which is a non-ionic surfactant; it has been reported that this surfactant does not affect the structural integrity of CLG and elastin [19], while alkaline surfactants affect the content of glycosaminoglycans [26]. The remaining DNA content in Dcell-BP was only 13.1 ± 1.6 ng/mg dry weight, so it can be considered as decellularized scaffold in according to Crapo et al., who has proposed that 50 ng/mg of residual DNA for a scaffold to be considered decellularized [43].

FTIR results showed that the spectrum of n-BP was very similar to the one of GC-BP, although subtle differences can be detected. Thus, a reduction in the intensity of the band at 3296 cm^−1^ as well as, an increase in signals located at 2922 cm^−1^ and 2851 cm^−1^ were observed after the modification process. As mentioned above, the first band has been related to N-H stretching vibration from amine functionalities (-NH_2_) existing in CLG structure of BP whereas the last band is due to asymmetric and symmetric C-H stretching vibrations of methylene groups. The decrease in intensity of the band at 3296 cm^−1^ after the crosslinking process is due that these groups reacts with GA to yield a Schiff base [46–49]. Similarly, the increase in the intensities of the bands of methylene groups was observed because a greater number of methylene groups belonging to the GA are presented in the GC-BP sample.

Regarding DSC measurements, an endothermic transition (136 °C) in n-BP has been previously reported by Bozec et al. [37] who attributed this event to evaporation of residual strongly H-bonded water responsible for the stability of the triple helix of CLG. This signal was shifted to higher temperatures when the BP was decellularized and crosslinked with GA as CG-BP sample showed this peak at 142 °C. This means the release of water is more difficult in crosslinked samples in comparison to non-treated sample. On the other hand, the endothermic peak at 226 °C (present in n-BP but absent in CG-BP), has been attributed to conformational changes of the CLG molecules from a triple helix structure to random coil [37,50,51]. This observation seems to indicate that the decellularized and crosslinked processes generate some conformational changes in CLG molecules.

TGA and DTGA curves for n-BP and GC-BP showed that the crosslinking process did not modify the thermal stability of BP, as other authors have suggested for BP and CLG [37,47,50]. These authors stated that the Td and the onset temperature of decomposition increase by increasing the CLG crosslinking with GA. Nevertheless, our results are not unusual and it has been also reported previously by Goissis et al. [47], who attributed it to a low GA concentration used during the crosslinking process. Both samples exhibited two mass losses located at 60 °C and 320 °C; the first one is due to the release of loosely bound water while the second one is attribute to the first stage bulk degradation of dried CLG fibril [37,50].

Mechanical properties results indicated that the CG-BP had lower tensile strength and Young modulus but higher deformation than that exhibited by n-BP. This trend is in agreement to reported by Mendoza-Novelo et al., who characterized BP crosslinked with GA in both orthogonal directions (0° y 90°) [52]. Olde Damink et al. also found a similar behavior when analyzing CLG based biomaterials [46]. In contrast, Duraipandy et al. reported the opposite behavior, i.e., the crosslinking process with GA increased the tensile properties of CLG [53]. Thus, mechanical properties results seem to support the hypothesis coming from DSC measurements, where it was postulated that conformational changes of the CLG molecules, from a triple helix structure to random coil, take place when BP are decellularized and crosslinked with GA, which led to lower mechanical properties.

*In vitro* characterization of n-BP and GC-BP in contact with DPSCs showed that the scaffolds have the ability to promote cell adhesion and cell viability greater than 70%; therefore, both scaffolds can be considered as biocompatible scaffolds in according ISO 10993-5 standard [54]. These results are in agreement with the work of Ferroni et al., who demonstrated that the treatment of BP with GA promoted cell adhesion [55]. Ahmadpour et al. observed the opposite behavior when 1% of GA was used to crosslink BP [14], however, in the present work 0.5% of GA was used. Moreover, GC-BP promoted an increase in cell viability in comparison to n-BP at 24 h of exposure with the scaffolds, which suggests that decellularization and GA crosslinking stimulate the cell activity. This finding is in agreement with works by Ferroni and Heuschkel [25,55] who demonstrated that decellularization of BP increases 70% the cell viability of human adipocytes stem cells versus native BP at 72 h. In addition live-dead assay showed that GC-BP promoted cell survival, as it was possible to observe a greater number of viable DPMSCs on the surface of GC-BP scaffold than that displayed on the n-BP scaffold. Since these dental cells have been shown to participate in tissue regeneration processes, and they can be stimulated to differentiate into osteoblasts, then the scaffold has the potential for application in guided bone regeneration. However, studies related to bone formation and osteogenic differentiation in presence of the scaffold should be performed.

## 5. Conclusion

The decellularization and crosslinking of BP appeared to induce conformational changes of the CLG molecules, which led to lower mechanical properties at the GC-BP scaffold, at the same time that promoted cell adhesion and viability of DPSCs, therefore, in this study we suggest that the decellularized and GA-crosslinked BP is a scaffold with the potential to be used in the oral cavity.
